# 

**DOI:** 10.1017/psy.2025.10061

**Published:** 2025-11-25

**Authors:** Geoffrey Blondelle

**Affiliations:** CRP-CPO, UR UPJV 7273, Université de Picardie Jules Verne, France

## 1 Introduction

The book *An Introduction to Psychometrics and Psychological Assessment* (2nd Edition) by Cooper (2023) provides a comprehensive and accessible guide to understanding, using, and developing psychological tests and assessments. This volume is the second edition of Cooper’s work, expanding on its predecessor—the prize-winning *Psychological Testing: Theory and Practice* (first published in 2018)—by incorporating a range of new methodologies and addressing emerging challenges in psychometric assessment. The second edition incorporates several significant updates, including enhanced treatment of measurement invariance, expanded coverage of fairness and bias detection, and the integration of contemporary methodologies, such as Internet-based testing and automatic item generation. Cooper, a well-established scholar in psychology and assessment, draws on his extensive teaching and research experience to deliver a volume that is both rigorous and accessible.

Designed for a broad audience, the book explicitly caters to advanced undergraduate and postgraduate students in psychology and related fields, and it is equally valuable for researchers, test developers, and practitioners in applied settings (e.g., in educational, clinical, or organizational contexts). A solid grounding in introductory psychology and basic statistics (e.g., correlation and regression) is recommended for readers to fully benefit from the book. The author notes its utility as a classroom text, but it is equally useful as a self-study resource for professionals updating their psychometric knowledge.

## 2 Content overview

The book is structured into 18 chapters (436 pages), each carefully designed to build from fundamental principles to advanced methods, while consistently addressing the implications of test use in real-world contexts. Cooper does not simply describe methods; he situates them historically, explains their assumptions, and critically evaluates their limitations. This reflective and practical approach ensures that readers understand both *how* to use psychometric tools and *why* certain choices matter.

*Chapters 1–3 (Foundations of Psychometrics):* These chapters introduce psychometrics as a discipline, its historical development, and its central role in psychology. Cooper clearly distinguishes between tests, scales, and questionnaires, highlighting the purposes and assumptions underlying each. He frames measurement as the translation of latent constructs into observable scores, emphasizing both its utility and its challenges. This perspective helps readers appreciate psychometrics not as a purely technical exercise but as a way of operationalizing psychological theory.

*Chapter 4 (Administering and Scoring Tests):* Here, Cooper stresses the importance of standardized administration and the risks of bias introduced by inconsistent procedures. He illustrates with examples how careless administration can undermine reliability and fairness, preparing readers to think critically about test use in practice.

*Chapter 5 (Interpreting Test Scores):* The discussion of norms, standard scores, and percentiles is enriched by Cooper’s insistence on transparency and caution in interpretation. He warns against over-simplifying test results and underscores the ethical responsibility of test users to communicate scores responsibly.

*Chapter 6 (Correlation):* Correlation is presented not just as a statistical tool but as the conceptual backbone of psychometric theory. Cooper carefully shows how correlations underlie reliability and validity evidence, guiding readers to see connections between statistics and the logic of measurement.

*Chapters 7 and 8 (Reliability and Generalizability):* Reliability is first explained in classical terms, including test–retest, internal consistency, and split-half methods. Cooper critiques traditional metrics, such as Cronbach’s alpha, referencing contemporary concerns about its limitations. He then expands to Generalizability Theory, illustrating how reliability can be decomposed across multiple sources of error. This nuanced treatment demonstrates Cooper’s pedagogical style: he introduces the “basic” measure, then challenges it, and finally provides more advanced frameworks to resolve its weaknesses.

*Chapter 9 (Validity, Bias, and Invariance):* A highlight of the book, this chapter reflects Cooper’s commitment to fairness and ethical assessment. He explains different forms of validity (content, criterion, construct) before moving to contemporary issues such as measurement invariance and differential item functioning (DIF). Importantly, he situates fairness as not just a technical matter but as a social and ethical imperative. The discussion of cultural bias and test translation, aligned with international guidelines, demonstrates Cooper’s forward-looking perspective on psychometric practice and is consistent with the Standards for Educational and Psychological Testing (AERA, APA, & NCME, 2014), which emphasize fairness as a central pillar of valid assessment.

*Chapters 10–13 (Factor Analysis):* These chapters form the book’s backbone for multivariate methods. Cooper begins with exploratory factor analysis (EFA), carefully showing readers how factors are extracted and rotated, and what conceptual meaning these choices carry. Chapter 11 deepens this with applied examples, including practical guidelines for interpreting factor loadings. Chapter 12 introduces alternative designs, such as confirmatory factor analysis (CFA), while Chapter 13 explores hierarchical models, highlighting their value in studying broad constructs like intelligence or personality. Across these chapters, Cooper’s emphasis is always on interpretation and application: he warns readers against “blindly trusting” statistical output and models, stressing the need for theoretical justification and critical thinking.

*Chapter 14 (Network Analysis):* This newly added chapter introduces readers to an emerging paradigm. Cooper highlights how network models conceptualize psychological variables as interrelated systems rather than latent traits. By providing examples from psychopathology and personality, he helps readers see how networks offer alternative perspectives, while acknowledging that the approach is still evolving and not without limitations. This reflects a broader movement in psychometrics toward network approaches (Epskamp et al., 2018), which emphasize the dynamic interplay among psychological components rather than their reduction to latent dimensions.

*Chapter 15 (Item Response Theory):* Cooper presents IRT in a non-technical, intuitive manner, explaining its advantages over classical methods (e.g., in adaptive testing and scale precision). He illustrates key concepts, such as item characteristic curves with simple examples, making IRT approachable while also noting its growing importance in large-scale assessment contexts.

*Chapter 16 (Test and Scale Construction):* This chapter provides step-by-step guidance on writing, piloting, and validating test items. Cooper emphasizes the iterative nature of test development and warns against common pitfalls, such as assuming Likert-scale totals always represent interval-level data. This critical stance encourages students to think carefully about design choices.

*Chapter 17 (Problems with Test Scores):* Here, Cooper invites readers to reflect critically on the limitations of psychometric scores. He discusses threats to validity, the dangers of over-generalizing from scores, and the potential misuse of assessments in applied contexts.

*Chapter 18 (Psychometrics in Context):* The book concludes with a reflective perspective, situating psychometrics within broader psychological, societal, and ethical debates. Cooper raises philosophical questions (e.g., are psychological attributes truly quantifiable?) and links psychometric practices to their consequences in education, health, and policy. This chapter demonstrates Cooper’s balanced perspective: psychometrics is powerful, but it is not neutral, and its use requires ongoing critical scrutiny.

## 3 Contribution of the second edition and pedagogical features

A standout feature of this textbook is its suite of the Supplementary Material, which significantly enriches the learning experience. In lieu of dense mathematical formulas in the main text, Cooper provides a series of simple-to-use Excel/OpenOffice spreadsheets that accompany most chapters. These spreadsheets (available for download from the publisher’s website at routledge.com/9781032146164) allow readers to actively engage with psychometric concepts. For instance, one spreadsheet performs a VARIMAX factor rotation, letting the user see how factor analysis works in practice; another simulates how test length affects reliability, vividly demonstrating the Spearman–Brown prophecy formula; yet another functions as an item-analysis tool that flags poorly performing items in a test. All spreadsheets are integrated with exercises in the book, with clear instructions and expected learning outcomes. By manipulating these interactive tools (e.g., changing values in designated cells and observing the outcome in real time), readers can experiment with psychometric principles and witness their effects, which deepens understanding. The second edition maintains and expands this hands-on approach. Cooper notes that the spreadsheets and other software referenced in the text can be freely downloaded from Routledge’s website, ensuring that readers have easy access to these resources. This is an excellent pedagogical strategy: it transforms what could have been dry textbook calculations into tangible, experiential learning. Students in a classroom setting, in particular, will find these resources invaluable for assignments and self-study. The inclusion of these tools sets the book apart from more conventional psychometrics textbooks and aligns with modern best practices for active learning.

## 4 Critical evaluation

Cooper’s book excels in clarity, organization, and pedagogy. Complex concepts are explained in plain language, often with real-world examples and even humor. A major innovation is the integration of downloadable Excel/OpenOffice spreadsheets, which allow readers to experiment with psychometric concepts, such as reliability, factor rotation, and item analysis. This experiential approach aligns with best practices in active learning and makes abstract ideas tangible. The breadth of coverage is impressive, from classical test theory to modern methods like IRT and network analysis. The emphasis on fairness and bias, as well as on cultural adaptation of tests, reflects current priorities in the field. Cooper also addresses neglected issues, such as the dangers of unexamined scoring assumptions, encouraging critical thinking.

As an introductory text, some cutting-edge areas—particularly AI and machine learning in psychometrics—are only hinted at through topics like automatic item generation. While understandable, future editions might expand more on computational approaches, adaptive testing, and ethical challenges in algorithmic assessment. Additionally, Cooper devotes space to philosophical debates, such as the “levels of measurement” (Stevens, 1946), questioning whether psychological constructs can be meaningfully quantified as interval or ratio variables. He draws on critiques from authors like Joel Michell, who argued that psychology may have adopted the rhetoric of measurement without demonstrating that psychological attributes are genuinely quantitative. These sections encourage readers to adopt a reflective stance and to consider the epistemological foundations of psychometrics. However, for some readers—particularly those seeking primarily practical guidance—such theoretical digressions may feel tangential. While not directly applicable to everyday test construction or validation, they nonetheless add intellectual depth and remind the reader that psychometric practice is embedded in broader philosophical debates.

## 5 Relevance and applications

The book is particularly valuable for advanced undergraduate and graduate students, educators in psychology and related disciplines, and practitioners in applied contexts. For students, its emphasis on practical skills ensures that knowledge can be directly applied in real-world settings—whether developing new assessments, conducting empirical data analyses, or interpreting test results. The discussions on cultural bias and best practices for test translation are especially timely in light of the globalization of psychological assessments.

For practitioners, the book provides clear frameworks for selecting and administering tests in clinical (e.g., psychological evaluation and diagnosis), educational (e.g., student assessment and guidance), and organizational (e.g., personnel selection and development) contexts. Its attention to ethical issues and test limitations further enhances its practical relevance.

## 6 Conclusion

Cooper’s *An Introduction to Psychometrics and Psychological Assessment (2nd Edition)* is a standout resource that bridges theory, practice, and ethics in testing. It offers comprehensive coverage of classical foundations and modern innovations, enriched by interactive pedagogical tools. While certain emerging areas (e.g., AI-driven psychometrics) remain beyond its scope, the book equips readers with the solid grounding necessary to approach those frontiers. In sum, this is an essential text for students, researchers, and practitioners seeking a rigorous yet accessible introduction to psychometrics. Cooper’s ability to make technical material both understandable and engaging ensures that the book will remain a leading reference for years to come.
